# FKBP52 overexpression accelerates hippocampal-dependent memory impairments in a tau transgenic mouse model

**DOI:** 10.1038/s41514-021-00062-x

**Published:** 2021-05-03

**Authors:** Marangelie Criado-Marrero, Niat T. Gebru, Lauren A. Gould, Danielle M. Blazier, Yamile Vidal-Aguiar, Taylor M. Smith, Salma S. Abdelmaboud, Lindsey B. Shelton, Xinming Wang, Jan Dahrendorff, David Beaulieu-Abdelahad, Chad A. Dickey, Laura J. Blair

**Affiliations:** 1 Department of Molecular Medicine, Morsani College of Medicine, USF Health Byrd Alzheimer’s Institute, University of South Florida, Tampa, FL USA; 2 Department of Molecular Pharmacology and Physiology, College of Medicine, USF Health Byrd Alzheimer’s Institute, University of South Florida, Tampa, FL USA; 3 Research Service, James A Haley Veterans Hospital, Tampa, FL USA

**Keywords:** Diseases of the nervous system, Cognitive ageing, Learning and memory

## Abstract

Abnormal accumulation of hyperphosphorylated tau induces pathogenesis in neurodegenerative diseases, like Alzheimer’s disease. Molecular chaperones with peptidyl-prolyl cis/trans isomerase (PPIase) activity are known to regulate these processes. Previously, in vitro studies have shown that the 52 kDa FK506-binding protein (FKBP52) interacts with tau inducing its oligomerization and fibril formation to promote toxicity. Thus, we hypothesized that increased expression of FKBP52 in the brains of tau transgenic mice would alter tau phosphorylation and neurofibrillary tangle formation ultimately leading to memory impairments. To test this, tau transgenic (rTg4510) and wild-type mice received bilateral hippocampal injections of virus overexpressing FKBP52 or GFP control. We examined hippocampal-dependent memory, synaptic plasticity, tau phosphorylation status, and neuronal health. This work revealed that rTg4510 mice overexpressing FKBP52 had impaired spatial learning, accompanied by long-term potentiation deficits and hippocampal neuronal loss, which was associated with a modest increase in total caspase 12. Together with previous studies, our findings suggest that FKBP52 may sensitize neurons to tau-mediated dysfunction via activation of a caspase-dependent pathway, contributing to memory and learning impairments.

## Introduction

The 52 kDa FK506-binding protein (FKBP52) is a ubiquitous immunophilin that is highly expressed in neurons particularly in brain areas like the hippocampus and cortex^1^. FKBP52 is mainly known for its chaperone and cis-trans peptidyl-prolyl isomerase (PPIase) activities. The PPIase activity of this immunophilin occurs via the FK1 domain where it interacts with other proteins, such as steroid hormone complexes^2,3^. There are multiple studies describing the role of FKBP52, in collaboration with the 90 kDa heat shock protein, Hsp90, as a major regulator of glucocorticoid receptors (GRs). Their association, along with a contribution from other PPIases, affects hormone binding and transactivation, which alters the expression of genes involved in cell survival and proliferation^2,4,5^. During this event, FKBP52 serves as an adaptor between dynein motor protein and the GR heterocomplex on microtubules, which may promote nuclear translocation^2,6,7^.

Molecular chaperones have diverse functions through multiple pathways in stress-related, reproductive, and immune disorders as well in neurodegenerative disease^7–10^. Alzheimer’s disease (AD) is a neurodegenerative disorder characterized by an abnormal accumulation and hyperphosphorylation of the microtubule-associated protein, tau^11–13^. Microtubules are an important cytoskeletal component of neurons by providing structural support. In addition, these filaments are known to be essential for the development and maintenance of neurons as well as for the transport and delivery of proteins to synapses (see review ref. ^14^) or the nucleus, as described above. Since tau stabilizes microtubules, the dysregulation of tau can impact neuronal health. FKBP52 is known to prevent microtubule formation by binding and removing tubulin from microtubules^15^. In addition to this, FKBP52 can induce aggregation of multiple tau species including wild-type tau, P301L tau (contains a proline-to-leucine mutation at position 301 as found in frontotemporal dementia), and a Tau-F4 fragment (a tau truncation with strong microtubule binding)^16–18^.

Overexpression of FKBP52 increased recombinant tau oligomerization and fibril formation, while preventing tau accumulation and reducing neurite outgrowth in cells^19^. In line with this, another study in a P301L-tau zebrafish showed restored axonal outgrowth and branching in spinal motoneurons when FKBP52 was knocked down, which led to an improvement in escape behavior^17^. There are also contradictory reports, which show FKBP52 increasing neurite outgrowth in hippocampal cells^20,21^. Taken together, these data indicate that FKBP52 can modulate tau and microtubule stability affecting neuronal differentiation.

To the best of our knowledge, the effects of FKBP52 and tau on learning and memory as well neuronal viability have not been investigated. Although several studies have reported that FKBP52 can induce changes in tau dynamics, further research is needed to understand if FKBP52 is able to promote tau oligomerization and affect neuronal toxicity in the brain. In this study, we report that overexpression of FKBP52 in the hippocampus of tau transgenic mice, rTg4510, leads to impairments in learning accompanied by long-term potentiation (LTP) deficits and hippocampal neuronal loss. Our findings suggest that FKBP52 sensitize neurons to tau-mediated damage-inducing neuronal loss and synaptic changes contributing to memory and learning impairments without affecting the overall levels of phosphorylated tau.

## Results

### FKBP52 induces tau aggregation and fibril formation in vitro

**Fig. 1:**
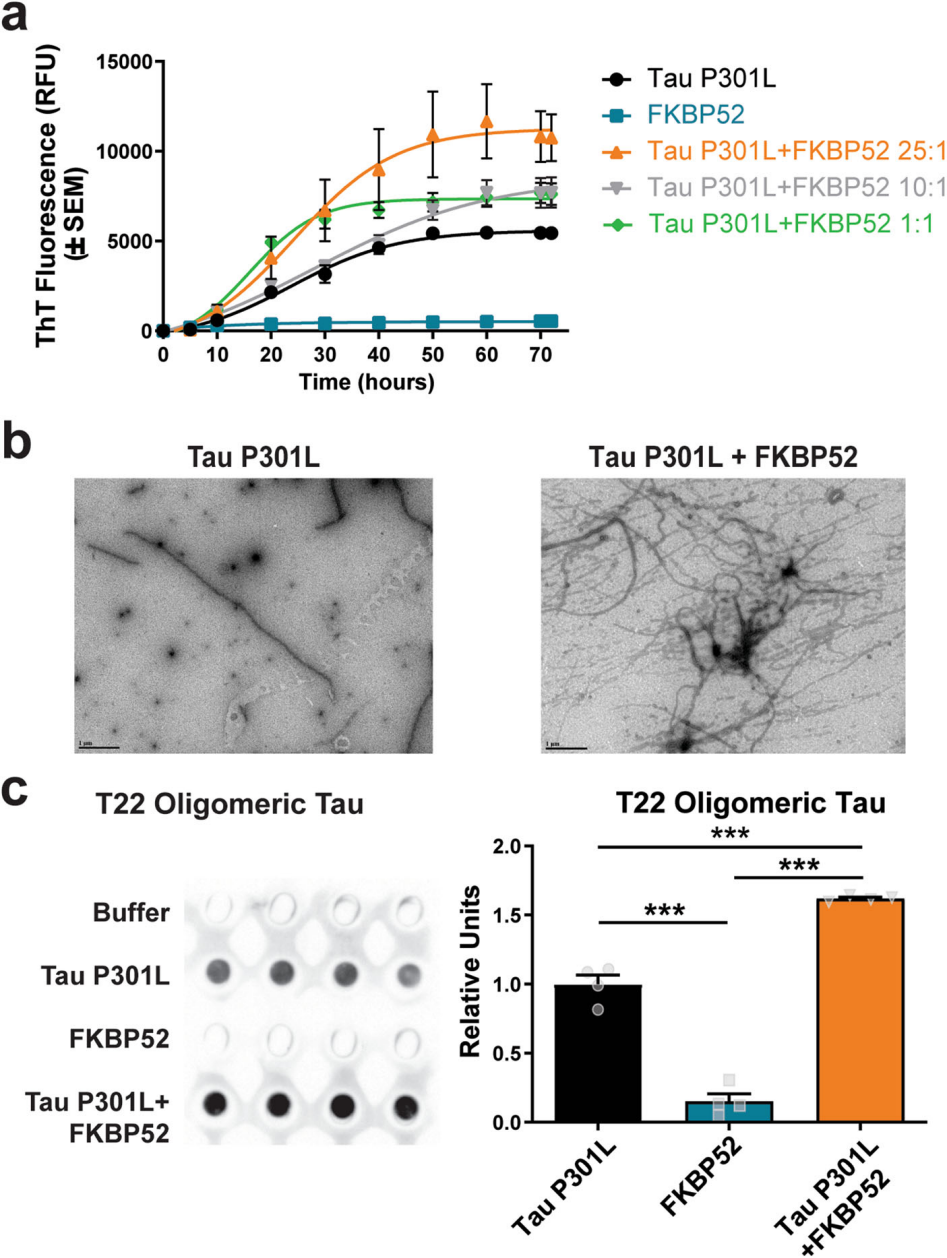
FKBP52 accelerates tau P301L protein aggregation. **a** Using a thioflavin T (ThT) fluorescence assay, tau P301L (20 μM) was monitored over 72 h with or without coincubation of FKBP52 (20, 2, or 0.8 µM). **b** Representative transmission electron microscopy (TEM) images are shown (×20,000 magnification; scale bar 1 μm). **c** Left panel: Representative dot blot analysis of tau aggregates incubated with 0.5 µg of tau P301L, 0.5 µg of FKBP52, or combination of (0.5 µg Tau and 0.5 µg FKBP52) probed with T22 antibody. Right panel: Quantification of T22 expression in dot blot with samples carried out in quadruplet. Results represented as standard error of the mean (±SEM); experiment was run in triplicate, **p < 0.001 by one-way ANOVA.

Since it has been shown that recombinant FKBP52 induces the formation of tau-P301L oligomers^17,22^, we first investigated the effect of this chaperone on tau protein aggregation dynamics and fibrils formation in vitro. We incubated recombinant FKBP52 with tau-P301L at different molar ratios [25:1, 10:1, and 1:1] to assess how protein concentration affects their interaction and influences tau aggregation over the course of 72 h (Fig. 1a). FKBP52 promoted tau aggregation as measured by Thioflavin T (ThT) fluorescence, a readout of β-sheet formation. These observations were confirmed using transmission electron microscopy (TEM), which revealed increased fibril formation when tau-P301L was incubated with FKBP52 (Fig. 1b). Then, we assessed the presence of tau oligomers by probing with T22 antibody^23^. In line with a previous study^17^, we determined that FKBP52 induces recombinant tau oligomerization (Fig. 1c). Overall, these findings validate previous reports showing that, independent of Hsp90, FKBP52 can induce tau aggregation into oligomers and fibrils, suggesting that FKBP52 may contribute to tauopathies by directly affecting tau assembly dynamics.

### Viral transduction induced FKBP52 overexpression in the hippocampus of rTg4510 mice

**Fig. 2:**
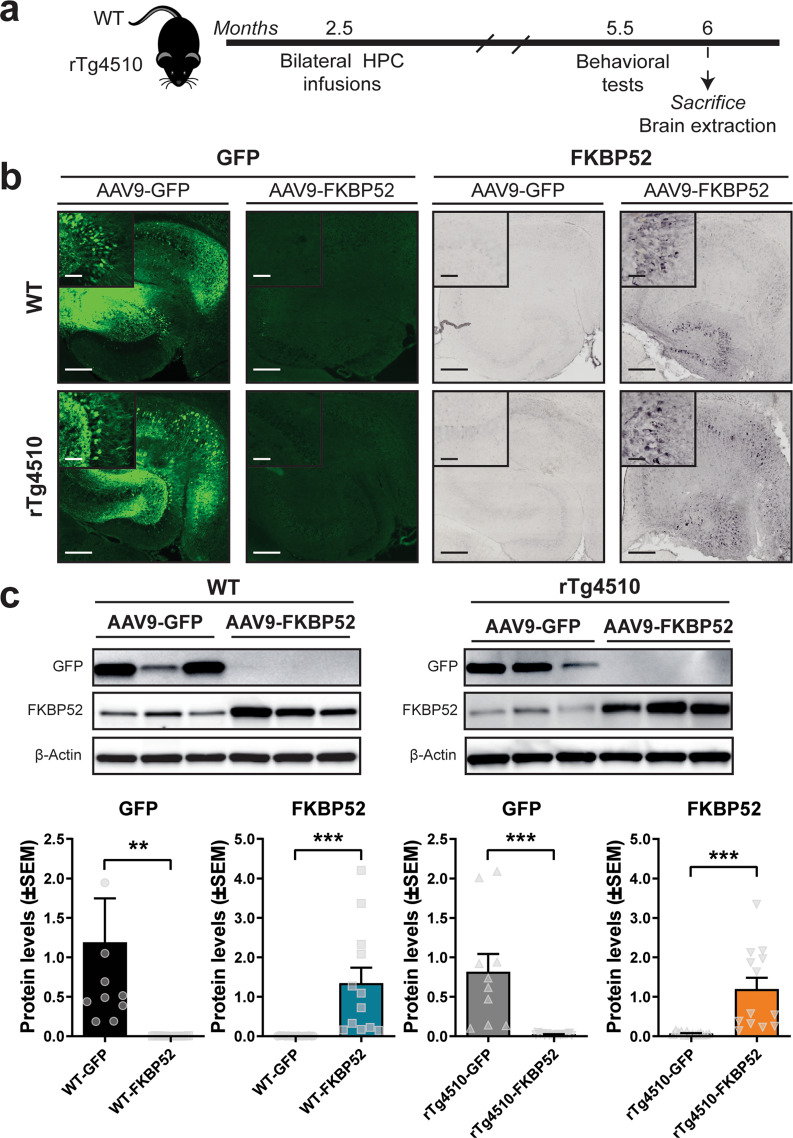
Experimental design and confirmation of GFP and FKBP52 overexpression in the hippocampus. **a** Timeline of bilateral hippocampal AAV injections and experiments performed in WT and rTg4510 mice. **b** Representative images of AAV9-GFP and AAV9-FKBP52 hippocampal sections after 3 months of viral expression. **c** Representative Western blots and quantification of GFP and FKBP52 overexpression in these animals. Wild-type mice [*n* = 16], rTg4510 mice [*n* = 16]. Protein levels were normalized to ß-Actin and analyzed by unpaired *t* test, where statistical significance is represented by **p < 0.01 and ***p < 0.001. Scale bar represents 200 µm; inset scale represents 20 µm. AAV9 adeno-associated virus serotype 9, GFP green fluorescent protein, WT wild-type, HPC hippocampus.

Next, we investigated the effects of FKBP52 overexpression in the brains of tau transgenic mice. The rTg4510 tau transgenic mouse model shows progressive tau accumulation in the brain, which starts as early as 2 months of age with severe neuronal loss by 6-months^24–26^. Since we expected FKBP52 to worsen tau outcomes, we decided to use 2.5-month-old rTg4510 and wild-type mice, which is after tau has started to accumulate and before noticeable neuronal loss. These mice received bilateral hippocampal viral injections of AAV9-FKBP52 or AAV9-GFP (control). After 3 months of AAV expression, mice were exposed to behavioral testing (Fig. 2a). Following behavioral testing, brains were collected and expression of FKBP52 and GFP was confirmed by immunohistochemical (Fig. 2b) and Western blot (Fig. 2c) analyses.

### High levels of FKBP52 did not affect anxiety or locomotion

**Fig. 3:**
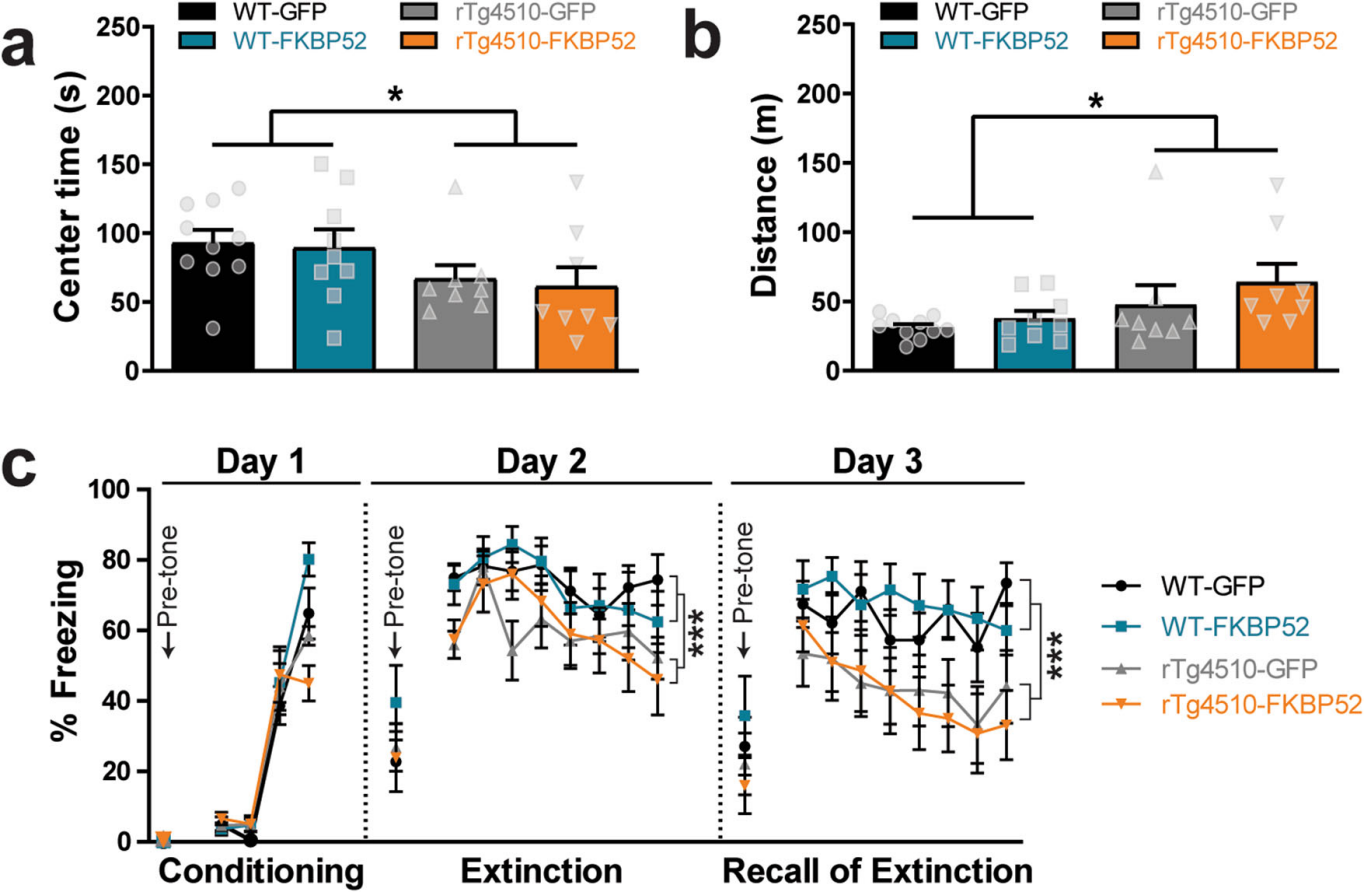
High levels of FKBP52 do not affect anxiety or locomotion in rTg4510 mice. **a** Anxiety levels and (**b**) total distance traveled were measured using the open field test. **c** Associative learning was evaluated using a fear conditioning and extinction tests. Percentage of freezing was calculated before (pre-tone) and during tone exposure. A total of 19 WT mice [AAV9-FKBP52, *n* = 9; AAV9-GFP mice, *n* = 10] and 16 rTg4510 mice [AAV9-FKBP52, *n* = 8; AAV9-GFP, *n* = 8] were used for these tests. AAV9, adeno-associated virus serotype 9, WT wild-type, s seconds, m meters, GFP green fluorescent protein. Data are shown in standard error of the mean (±SEM) and analyzed by two-way ANOVA (anxiety and locomotion) and repeated measures two-way ANOVA (fear); *p < 0.05, ***p < 0.001.

Since other studies have observed increased anxiety in mice lacking one allele of FKBP52 (FKBP52+/−)^27^, we first investigated this phenotype. Using the open field test, we found that wild-type mice spent significantly more time in the center (Fig. 3a), which is associated with lower anxiety levels. However, this was independent of FKBP52 or GFP expression. In line with previously studies^28^, tau transgenic mice displayed increased hyperactivity when compared to wild-type (Fig. 3b). We then evaluated associative fear learning and memory in these mice, because patients with AD have shown decreased ability to remember novel associations^29–31^. To test this, animals were exposed to fear conditioning (day 1), fear extinction (day 2), and recall of fear extinction (day 3) paradigms (Fig. 3c). Only during conditioning phase, there was a significant interaction between genotype and treatment (*p* < 0.005). However, following a two-way ANOVA, a Bonferroni post hoc test revealed that this difference was between rTg4510-FKBP52 and WT-FKBP52 where rTg4510 mice overexpressing FKBP52 showed impaired fear learning as measured by the freezing time during the tone exposure (*p* < 0.001). During extinction and recall of extinction, fear expression was significantly lower in rTg4510 mice (*p* < 0.001) when compared to wild-type mice, indicating an effect by genotype that was independent of the overexpressed protein (GFP or FKBP52). Only associative learning as measured during fear conditioning was slightly affected, suggesting that FKBP52 may influence the acquisition of new information when tau levels are elevated. It is possible that the hyperactivity characterizing these animals contributed to the lack of freezing during this task. Overall, we show that anxiety, locomotion, and associative memory were not altered in tau transgenic mice overexpressing FKBP52.

### Overexpression of FKBP52 in the hippocampus impaired spatial learning and working memory in rTg4510 mice

**Fig. 4:**
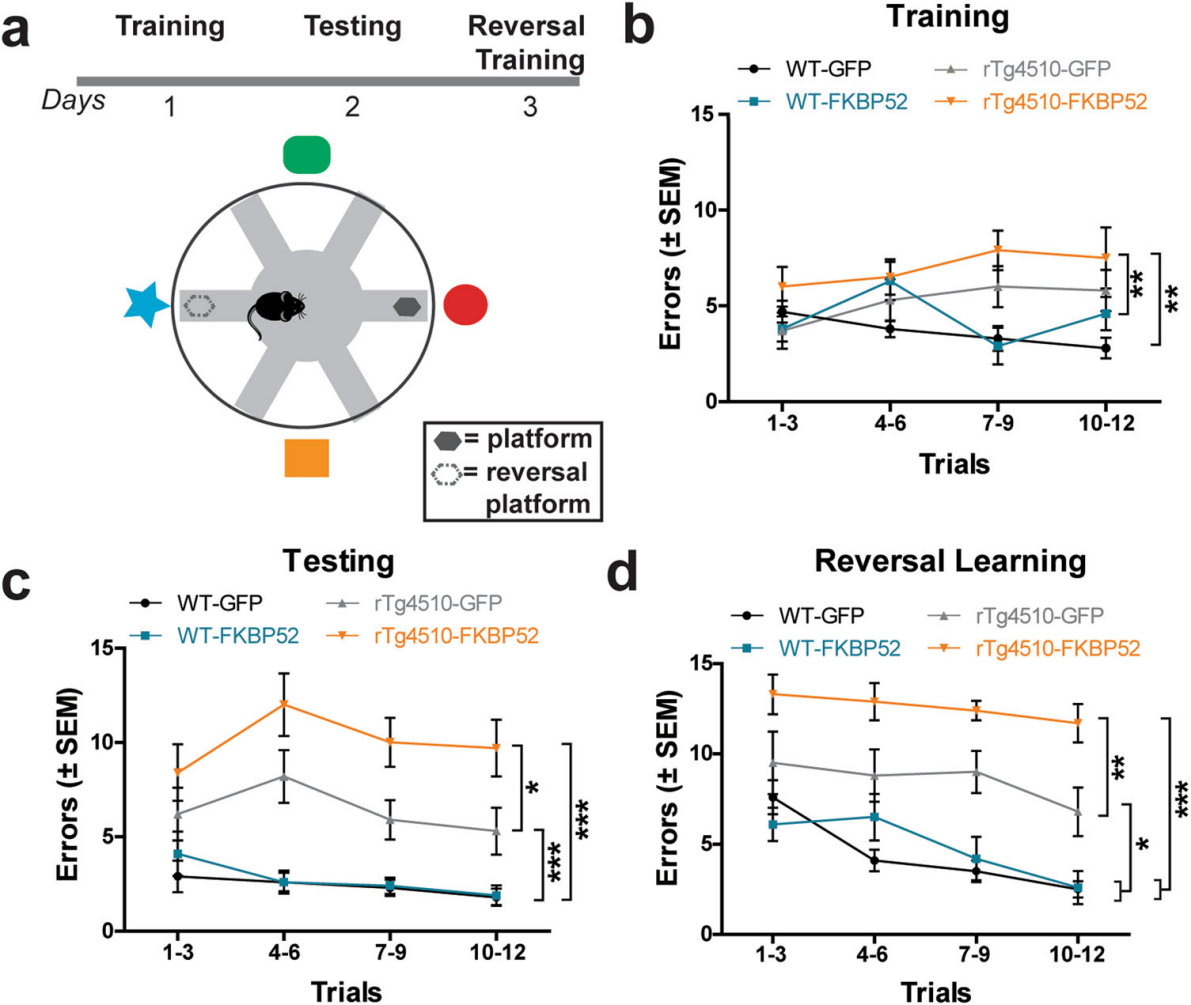
Overexpression of FKBP52 impairs spatial learning and working memory in rTg4510 mice. **a** Schematic of experimental protocol for the radial-arm water maze (RAWM) test which was used to evaluate spatial learning and working memory. The total number of errors to locate the platform was counted as the performance for each day. Each data point represents an average of 3–60 s trials. **b** On day 1, spatial learning (training), mice were trained for 12 trails alternating between visible and hidden platform. **c** On day 2, spatial memory (testing), platform remained hidden during all trials. **d** On day 3, for the reversal learning, the goal arm (reversal platform) was in the opposite arm to day 1. We used a total of 18 rTg4510 mice [*n* = 8 for AAV9-GFP; *n* = 10 for AAV9-FKBP52] and 19 wild-type mice [*n* = 10 for AAV9-GFP; *n* = 9 for AAV9-FKBP52] for this test. Data were analyzed by repeated measures two-way ANOVA followed by Bonferroni post hoc test (*p < 0.05, **p < 0.01, ***p < 0.001). Data are shown in standard error of the mean (±SEM). AAV9 adeno-associated virus serotype 9, WT wild-type, GFP green fluorescent protein.

We then used the radial-arm water maze (RAWM) task (Fig. 4a) to evaluated if FKBP52 overexpression in the hippocampus affected spatial learning and memory in rTg4510 and wild-type mice, as this is known to be altered in the rTg4510 model and is commonly disrupted in AD patients (reviewed in^32^). During training, FKBP52-overexpressing rTg4510 mice had a similar number of errors compared to GFP-injected rTg4510 (*p* > 0.05) (Fig. 4b). However, the number of errors was significantly higher when comparing rTg4510-FKBP52 to both wild-type groups (*p* < 0.05). Twenty-four hours later, mice received additional trials testing for memory deficits. On this day, memory impairments in rTg4510-FKBP52 mice were evident by significantly increased errors in locating the escape platform compared to rTg4510-GFP (*p* < 0.05), WT-GFP (*p* < 0.001), and WT-FKBP52 (*p* < 0.001) mice (Fig. 4c). We then increased task difficulty and tested the effects on learning flexibility by relocating the escape platform in the opposite arm (new goal) (Fig. 4d). Once again, overexpression of FKBP52 in rTg4510 mice led to the highest number of errors across trials, suggesting a lack of cognitive adaptation. No significant differences between FKBP52- or GFP-overexpressing wild-type mice were measured. Although both the rTg4510-FKBP52 and rTg4510-GFP groups showed deficits in learning and memory, clearly the ability to learn and recall was further disrupted in the tau transgenic mice when FKBP52 was overexpressed.

### FKBP52 overexpression induced deficits in hippocampal LTP

**Fig. 5:**
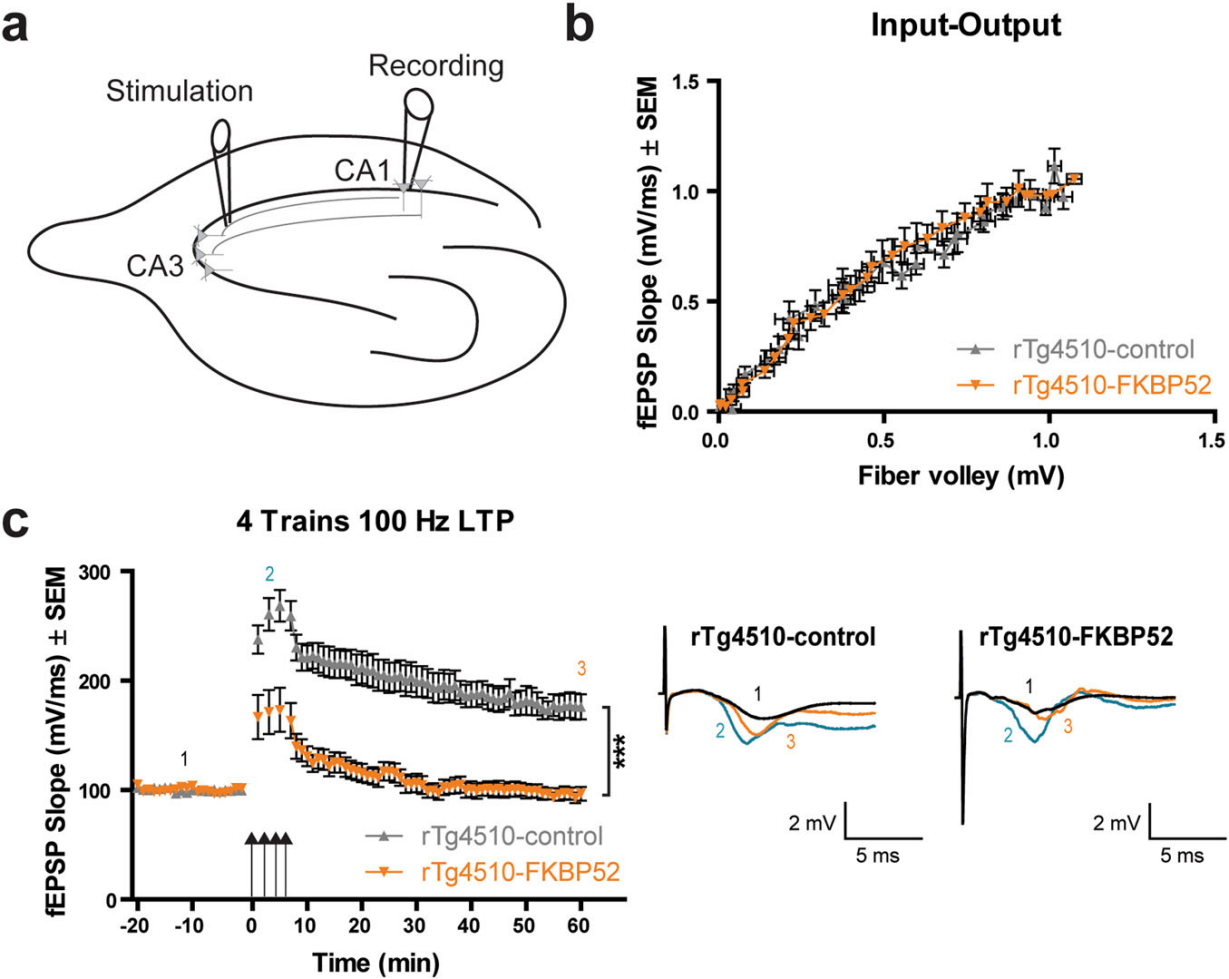
FKBP52 overexpression induces deficits in hippocampal LTP. **a** Schematic of the long-term potentiation (LTP) protocol in ex vivo hippocampal slices from rTg4510 mice infused with AAV9-control or AAV9-FKBP52. Stimulating electrode was positioned in the Schaffer collaterals (between CA3 and CA1) and recordings were obtained in the CA1 pyramidal neurons. **b** Input–Output (I–O) curve to evaluate pre- and postsynaptic excitatory function. The I–O curve compares the fEPSP slope (mV/ms) versus the fiber volley amplitude (mV). **c** Four-trains of high-frequency stimulation (HFS) at 100 Hz were given to induce LTP (indicated by the arrows). Following this, field EPSPs were measured for 60 min. Representative traces are shown on the right. Significance was determined by repeated measures two-way ANOVA (***p < 0.001). Data are shown in standard error of the mean (±SEM). *n* = 23 for rTg4510-control and *n* = 12 for rTg4510-FKBP52. Representative traces are shown as 1 (black) indicates baseline, 2 (teal) indicates initial early LTP in the first minute following HFS, and 3 (orange) indicates late LTP in the last 60 min of recording. fEPSP field excitatory postsynaptic potential, min minutes, CA1 CA3 Cornu Ammonis 1, 3, LTP long-term potentiation, mV millivolts, ms milliseconds, Hz hertz.

Long-term potentiation (LTP) is known to be affected in patients with AD as well as in rTg4510 mice^33,34^. Disruption of synaptic plasticity correlates with a more severe cognitive decline. Therefore, we evaluated if learning and memory impairments observed in the RAWM task were due to deficits in LTP. To test hippocampal LTP, we measured the field excitatory postsynaptic potentials (fEPSPs) through extracellular field recordings in ex vivo slices. The stimulation electrode was placed in CA3 (Schaffer collaterals) and the recording electrode was placed in CA1 (pyramidal neurons) (Fig. 5a). We measured the relationship between the excitatory input to CA3 neurons and the probability CA1 neurons will fire an action potential, better known as synaptic input–output (I-O) function. We show that FKBP52 did not alter this neuronal property in rTg4510 or control mice (Fig. 5b and Supplementary Fig. 1a). We also observed that the slope of fEPSPs (Fig. 5c) in rTg4510-FKBP52 mice was significantly reduced following high-frequency stimulation (HFS-represented by the arrow in Fig. 5c) compared to control transgenic mice. FKBP52 did not alter LTP in WT mice (Supplementary Fig. 1b). Altogether, these findings suggest that FKBP52 can further accelerate tau-mediated reductions in the ability to induce and maintain LTP.

### FKBP52 overexpression increased total tau without affecting tau tangles or phosphorylated tau species in the hippocampus of rTg4510 mice

**Fig. 6:**
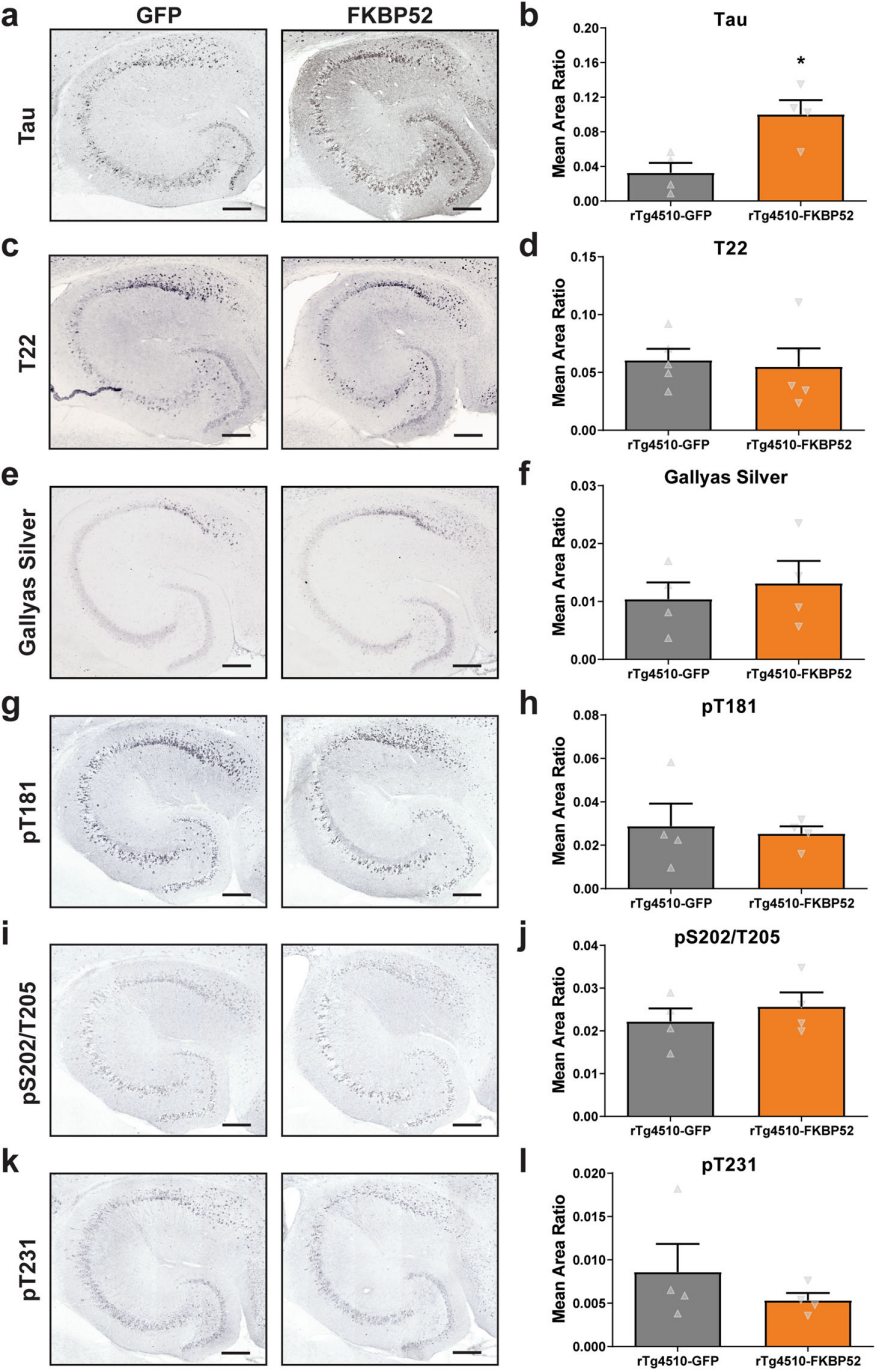
FKBP52 overexpression in rTg4510 mice does not alter phosphorylated tau. Tissue from AAV9-GFP and AAV9-FKBP52 injected rTg4510 mice was stained and analyzed for (**a**–**b**) total tau (DAKO), (**c**–**d**) T22, (**e**–**f**) Gallyas silver, (**g**–**h**) pT181 tau, (**i**–**j**) AT8 antibody recognizing tau phosphorylation at Ser202/Thr205 sites, and (**k**–**l**) AT180 antibody recognizing tau phosphorylation at Thr231 site. Protein levels were analyzed by unpaired *t* test, where statistical significance is considered by *p < 0.05 Results represented as standard error of the mean (±SEM); GFP, *n* = 4; FKBP52, *n* = 4;). Scale bars are 200 μm in the hippocampal tissue sections. GFP green fluorescent protein.

**Fig. 7:**
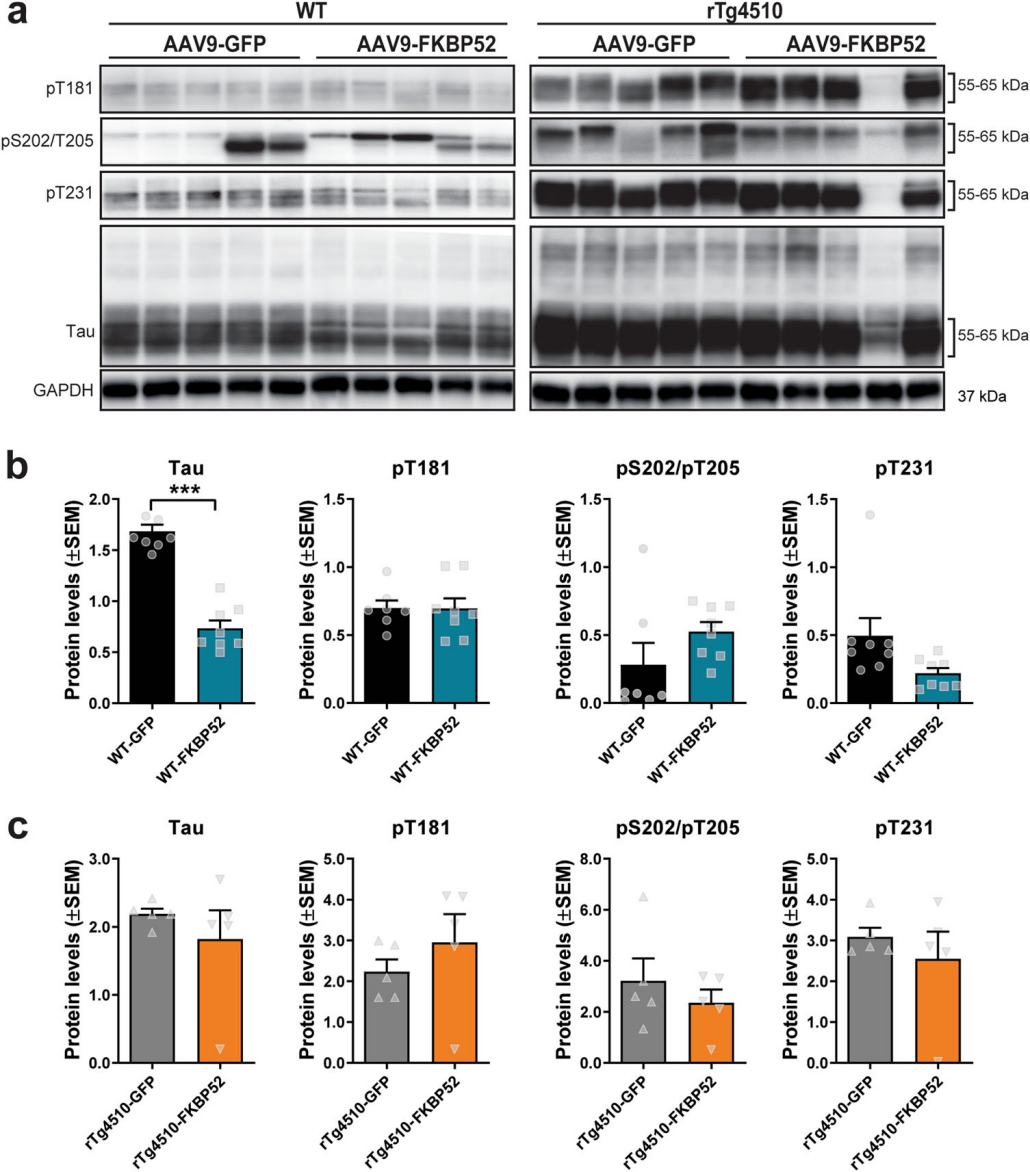
FKBP52 overexpression differently affects tau levels in WT and rTg4510 mice. **a** Representative Western blot analysis of soluble fraction from hippocampal tissue of WT and rTg4510 mice expressing either AAV9-GFP or AAV9-FKBP52. **b**, **c** Quantification of Western blots of soluble total (amino acids 1–150), pT181, pS202/pT205 (AT8), and pT231 (AT180) tau in WT and rTg4510 mice. All proteins were normalized to GAPDH. Results are were analyzed by unpaired *t* test and represented as the standard error of the mean (±SEM). WT-GFP (*n* = 8), WT-FKBP52 (*n* = 8), rTg4510-GFP (*n* = 5), rTg4510-FKBP52 (*n* = 5). kDa kilodalton, AAV9 adeno-associated virus serotype 9, GFP green fluorescent protein, WT wild-type, GAPDH Glyceraldehyde 3-phosphate dehydrogenase.

Since tau oligomers have been shown to inhibit LTP and memory formation in a human tau transgenic model (hTau)^35^, we examined whether LTP and memory deficits in rTg4510 mice were due to these tau structural changes in rTg4510 mice. We also evaluated whether FKBP52 affected the phosphorylation, solubility, or overall accumulation of tau in these mice. Total tau levels were significantly increased in rTg4510-FKBP52 mice (Fig. 6a, b). Despite this increase in total tau, the levels of oligomeric tau probed with T22 (Fig. 6c, d) and Gallyas silver-positive tau tangles were not affected by FKBP52 overexpression (Fig. 6e, f). Likewise, levels of tau phosphorylation at pT181 (Fig. 6g, h), pS202/T205 (Fig. 6i, j), and pT231 (Fig. 6k, l) sites were not altered. We further analyzed hippocampal subregions for levels of tau oligomers, using T22 antibody, and phosphorylation (pS202/T205), which also showed no detectible changes (Supplementary Fig. 2). We compared histological analyses to the expression of these proteins in the Western blot (representative images are shown in Fig. 7a). High expression of FKBP52 reduced total tau in wild-type mice (Fig. 7b), but no differences were detected in rTg4510 mice (Fig. 7c). Overall, these data suggest that FKBP52-induced deficits in cognition and synaptic plasticity were independent of tau oligomerization and phosphorylation.

### Overexpression of FKBP52 increases neurotoxicity in tau transgenic mice

**Fig. 8:**
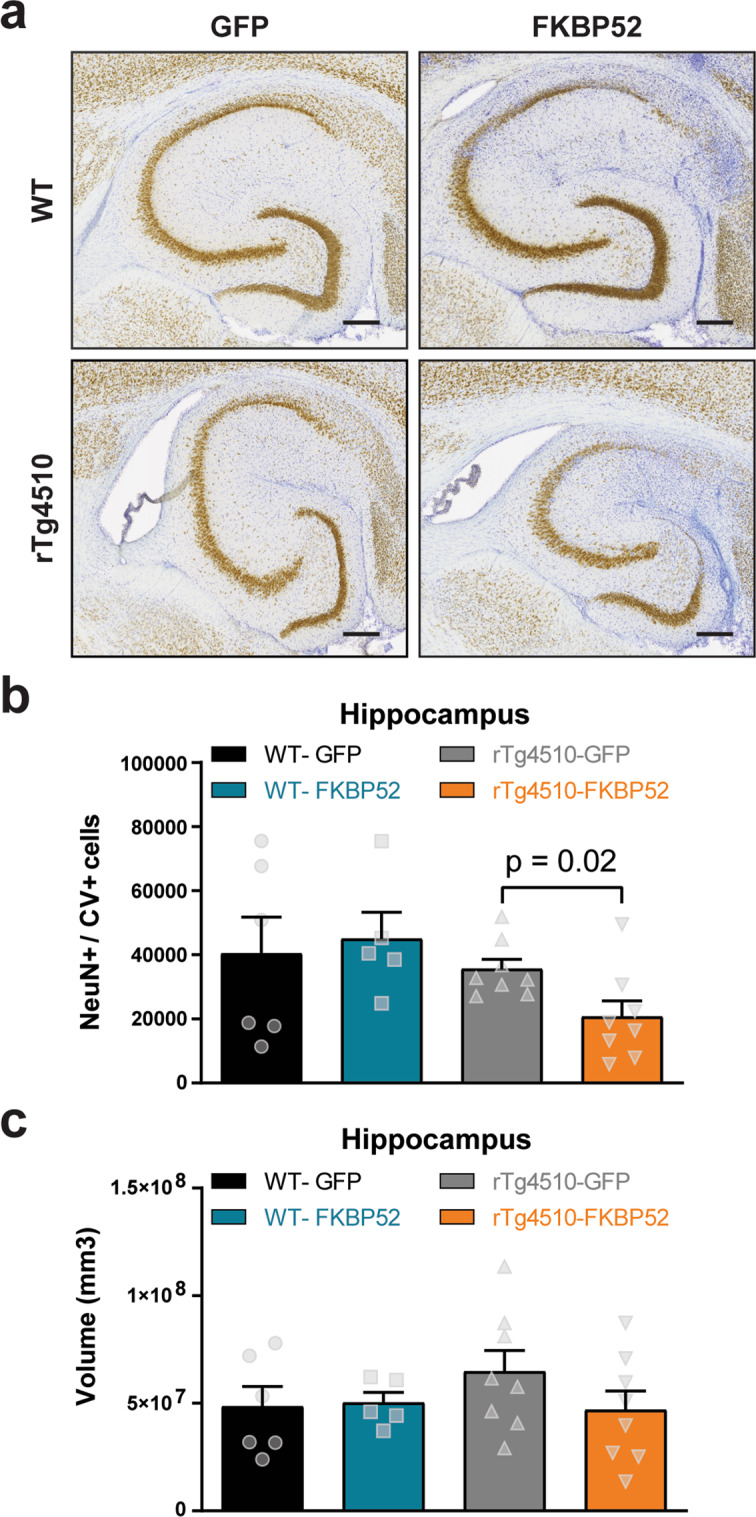
FKBP52 significantly affects neuronal density in tau transgenic mice. **a** Representative images from hippocampal neurons stained with NeuN (brown) and cresyl violet (CV, purple). These hippocampal slices correspond to AAV9-GFP and AAV9-FKBP52 injected mice. **b** Quantification of unbiased stereology from these animals. Results represent the standard error of the mean (±SEM). Data were analyzed by a two-way ANOVA and Bonferroni tests. Statistical difference in neuronal loss between rTg4510-GFP (*n* *=* 8) and rT4510-FKBP52 (*n* *=* 8) mice was evaluated by an independent *t* test (*p* *=* 0.02). **c** Hippocampal volume quantified by unbiased stereology. WT-GFP (*n* *=* 6) and WT-FKBP52 (*n* *=* 5). Scale bars are 200 μm. GFP green fluorescent protein, WT wild-type, mm^3^ cubic millimeter.

**Fig. 9:**
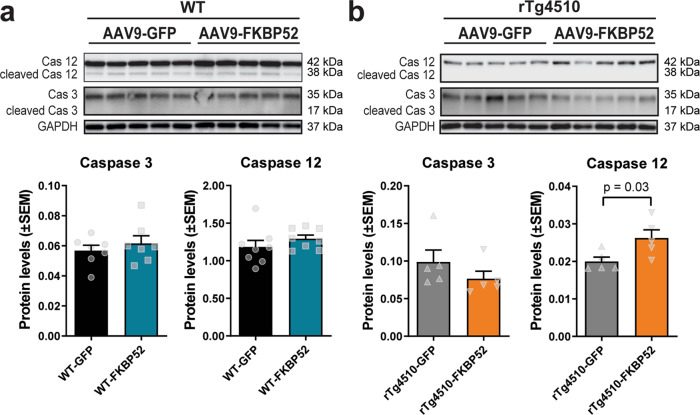
FKBP52 induces caspase 12 in rTg4510, but not WT, animals. **a** Caspase 3 and caspase 12 levels in WT mice injected with AAV9-GFP and AAV9-FKBP52. **b** Quantification of caspase 3 and caspase 12 in rTg4510 mice receiving hippocampal injections of the same virus. Proteins were normalized to GAPDH. Data were analyzed by Student’s *t* test and represented as standard error of the mean (±SEM). WT-GFP (*n* = 8), WT-FKBP52 (*n* = 8), rTg4510-GFP (*n* = 5), rTg4510-FKBP52 (*n* = 5). Cas caspase, kDa kilodalton, AAV9 adeno-associated virus serotype 9, GFP green fluorescent protein, WT wild-type, GAPDH Glyceraldehyde 3-phosphate dehydrogenase.

Another possibility is that FKBP52 may be affecting cognition and LTP through altering neuronal health^13^. After staining brain slices with NeuN and cresyl violet, we used unbiased stereology to investigate whether FKBP52 can induce hippocampal neuronal loss (Fig. 8a). The rTg4510 mice showed a significant reduction in neurons compared to WT mice (two-way ANOVA, *p* < 0.05; Fig. 8b). Because the number of samples in this analysis are low, we conducted an independent *t* test analysis revealing that rTg4510-FKBP52 had significantly less neurons than rTg4510-GFP mice (*p* < 0.01). However, hippocampal volume remained unchanged (Fig. 8c). We then tested whether neurotoxicity was driven by two regulators in the caspase signaling. We first examined caspase 3, which is known to mediate tau cleavage and levels correlate with cognitive deficits in aged mice^36^. Cleaved caspase 3 was not detected. Despite that caspase 3 levels were unchanged in WT and rTg4510 animals (Fig. 9a, b), levels of the proinflammatory caspase 12 were increased in rTg4510-FKBP52 mice (Fig. 9b). Although further research is needed to establish a functional role of FKBP52 on neurotoxicity, these findings suggest that neurotoxicity is enhanced in the presence of FKBP52 and P301L pathological tau.

## Discussion

FKBP52 was previously shown to induce tau aggregation and fibril formation in vitro; however, the effect of this chaperone in the brain and how it affects learning and memory was previously unknown. In this study, we fill this gap by investigating FKBP52 overexpression in vivo. We found that increased expression of FKBP52 in the hippocampus led to memory impairments in 6-month-old tau transgenic mice. These cognitive impairments were accompanied by hippocampal LTP deficits and neuronal loss, indicating that FKBP52 may influence neuronal health and function. Tau has been shown to be important for LTP, in fact, LTP deficits have been previously shown in mice lacking tau^37^ as well as mice overexpressing mutant tau^38^. Our study extends these findings by demonstrating that FKBP52 can potentiate this LTP deficit, further impairing cognition in rTg4510 mice.

We first wanted to confirm prior results demonstrating that FKBP52 promotes tau aggregation^16,17^. Using ThT and TEM, we supported and built upon these prior findings. Our results also indicate that FKBP52 does not self-aggregate. In addition, we show that high levels of FKBP52 in the hippocampus of wild-type mice did not impair learning or memory (Figs. 3, 4). Together these results suggest that both FKBP52 and tau are likely to cause the adverse cognitive effects observed. The possibility of altered FKBP52 levels inducing these effects by itself was also evaluated by an independent study, which showed that FKBP52 knockout mice do not have altered cognition^27^. These animals, FKBP52−/− and FKBP52+/+, were exposed to a series of behavioral tests assessing fear learning as well spatial and recognition memory at different ages ranging from 2 through 18 months of age. Investigators observed only motor coordination problems in FKBP52+/− mice without any changes in locomotion, anxiety, or memory. In our study, it is possible that overexpression of FKBP52 promotes activation of GR signaling, inducing similar effects to long-term stress or high levels of glucocorticoids exposure. Under both situations, stress and glucocorticoids, hippocampal synaptic plasticity and memory are impaired^39,40^. This correlates with our observations in rTg4510-FKBP52 mice presenting LTP and spatial memory deficits. Since the overexpression of FKBP52 in the hippocampus did not affect learning in WT mice, these data suggest that tau accumulation may increase vulnerability of stress-mediated defects and that GR signaling is likely not the only factor affecting LTP formation.

Another distinct property of FKBP52 is its potential participation in neurodifferentiation and neurodegeneration. Results from an in vitro study showed that neurite outgrowth was favored by FKBP52 overexpression in Neuro-2a (N2a) and embryonic hippocampal cells^20,21^. It is possible that FKBP52 neuroprotective actions identified in this study are through the induction of the nuclear factor k-light-chain-enhancer of activated B cells, NF-κB, which is known to regulate the expression of genes involved in several neuronal processes including synaptic plasticity as well as cell growth and differentiation^41^. Nevertheless, NF-κB is also a mediator of immune and inflammatory responses. Under uncontrollable stress, NF-κB can maintain a feed forward loop by inducing proinflammatory cytokines, which have been suggested to be implicated in the development of neurodegenerative disease (reviewed in ref. ^42^). Interestingly, upregulation of NF-κB and other genes involved in inflammatory pathways have been observed in rTg4510 mice as early as 2-months of age^43^. This suggests that FKBP52 may exert proinflammatory, and not neuroprotective, functions in the present study using a tau model, since high levels of FKBP52 and tau in the hippocampus resulted in neuronal loss.

Over the years, multiple therapies have been proposed for AD including reduction of tau^44–46^. Current advances include the development of positron emission tomography (PET) tracers for tau imaging, passive immunotherapy, tau active vaccines, antisense oligonucleotide therapies as well as tau aggregation inhibitors (reviewed in^47^). However, potential therapeutic efficacy and side effects are still being evaluated^13,47^. While our study highlights a possible mechanism where high levels of tau and FKBP52 generate cognitive impairments, there still many functional aspects of FKBP52 remain to be elucidated before its consideration as a potential therapeutic target for tauopathies. This is because ambiguous results in amyloidogenic pathway after modulating FKBP52 still remain. In a Aβ transgenic Drosophila model, FKBP52 overexpression reduced the Aβ-induced toxicity while increasing lifespan of these flies^48^. Although this has not been reported in mammals, conflicting results in Aβ and tau may not be beneficial to halt AD pathogenesis. In contrast, knocking down FKBP52 may be valuable for neural health as it induces extension in neurites and tubulin polymerization^15^. The authors proposed that binding of FKBP52 to tau may interfere with its association with tubulin preventing microtubule formation and stabilization. Thus, reducing its expression will preserve microtubule structure and dynamics. However, this benefit may not translate directly to behavioral changes in animals. For example, three-month-old FKBP52 + /− mice displayed anxiety-like behavior while, in a separate study, this was not observed in 18-month-old animals^27,49^. While these studies did not use a tauopathy model, the behavioral changes observed were still dependent on age and genotype.

Moreover, FKBP52 expression is lower in postmortem AD brains compared to controls, and its expression inversely correlated with the presence of neurofibrillary tangles (NFTs)^1,50^. Although tau tangles can be detected at 5.5 months of age in the rTg4510 mice, FKBP52 overexpression did not accelerate their formation, nor did it affect any of the tau phosphorylation sites that we probed. The most noticeable effect of FKBP52 on tau was in the induction of total tau levels, mainly in the CA3 hippocampal region, however, this change was not reflected in the Western blot analysis. It is possible that the rTg4510 model was too aggressive to be able to detect these changes, and that future studies at earlier timepoints or in less aggressive models may reveal tau-specific changes more clearly. Our results do suggest that activation of caspase signaling could be responsible for the FKBP52-induced neurotoxicity in tau transgenic mice. Based on previous findings, caspase 12 mediates cell death by inducing the endoplasmic reticulum stress pathway and by inhibiting proteasome activity, which then contributes to amyloid-beta toxicity^51,52^. More recent findings support the role of caspase-12 in inflammatory responses mainly via an activation of inflammasome or by modulation of other inflammatory caspases^53^. Additional research will be needed to test whether neuronal loss in rTg4510 mice is contingent on caspase 12 increase and if this can be prevented by blocking this protease, thus confirming a true dependence of this pathway. It is good to mention that it was recently demonstrated that other genes may contribute to the phenotype observed in our experimental model, rTg4510 mice. The insertion of tau and tTA transgenes, found in rTg4510 mice, disrupted other genes including the fibroblast growth factor 14 (*Fgf14*)^54^. These authors showed that tTA activity alone can contribute to neuron loss and LTP deficits as these mice can have weakened synaptic potentiation as early as two months of age^55^. However, we only see significant changes, neuronal loss and LTP, when FKBP52 is overexpressed suggesting an independent role for FKBP52 in this model. Certainly, further studies are needed to understand if there is interaction of FKBP52 with the affected genes, the contribution of FKBP52 at different time points in tau accumulation as well as to determine how the absence of FKBP52 affects tau. Based on the data from this study, it is possible that the decrease in FKBP52 expression in AD patients is due to the death of cells that have high FKBP52 levels during tau pathogenesis. Another remaining question is whether other brain regions as well as cell types are specifically vulnerable to FKBP52-mediated neurotoxicity and synaptic plasticity deficits or if wild-type mice become vulnerable to FKBP52-mediated effects as they age.

In conclusion, we found that FKBP52 by itself did not affect hippocampal neurons or cognition, but high levels of FKBP52 in combination with tau induced detrimental effects in spatial learning and memory while inducing caspase signaling in the rTg4510 tauopathy mouse model. Cognitive deficits did not correlate with tangle formation or phosphorylation of tau (pT181, pS202/205, pT231). Since progression of tau pathology is age-dependent, we should not exclude the possibility of FKBP52 influencing tau dynamics at later stages. Collectively, these findings support the involvement of FKBP52 in tauopathies by affecting cognition, synaptic plasticity, and neuronal viability.

## Methods

### Protein Expression

Protein expression and purification were performed as described previously^56^. Briefly, the recombinant human tau P301L and FKBP52 were cloned into a pET28A expression vector with six histidine (6XHis) tag and tobacco etch virus (TEV) sequence. Plasmids were transformed into *Escherichia coli* (BL21) one-shot star cells (Thermo Fisher Scientific C601003) a kanamycin-agar plate, purified, and dialyzed followed by a Coomassie staining purification and protein confirmation.

### Thioflavin T (ThT) fluorescence assay

Tau P301L (20 µM) was incubated with FKBP52 at a 25:1, 10:1, and 1:1 ratio in a 100 mM sodium acetate buffer (pH 7). Heparin (2.5 μM), DTT (2 mM), and thioflavin T (10 µM) were added for a total of 100 µL volumes in a black with clear bottom 96-well plate (Thermo Fisher Scientific 07-200-525). Fluorescence was read at 440 nm excitation and 482 nm emission in a BioTek Synergy H1 (BioTek Instruments, Inc., Vermont, U.S.A) plate reader over a period of 72 h. Experimental conditions were run in triplicates.

### Transmission electron microscopy (TEM) images

Protein samples (10 µL) were collected from the ThT assay and applied to copper grids (EMS300-Cu) for 1 min. After washing with deionized water (10 µL), excess water was absorbed using a filter paper. Uranyl acetate (1%) was applied for 30 s to stain samples. After drying overnight, images were captured with Gatan Orius wide-field camera using a JEOL 1400 Digital Transmission Electron Microscope (JEOL Ltd., Peabody, MA, USA). Fields shown are representative.

### Western Blot and Dot Blot Analysis

For the dot blot analysis, P301L and FKBP52 recombinant proteins were aggregated in 100 mM sodium acetate buffer for 72 h. A total of 0.5 µg of protein was spotted into nitrocellulose membrane. The membrane was submerged in 5% nonfat milk in TBST (blocking solution) followed by its development using ECL (Pierce #32106) on a LAS-4000 mini imager (General Electric Healthcare, Chicago, IL, USA). For mouse brain samples, tissue was homogenized in Hsaio TBS buffer, including protease inhibitors, followed by Sarkosyl-insoluble fractionation. Soluble proteins (S1, soluble 1 fraction) were run in a 4–15% SDS gradient gels (BioRad Laboratories Inc., Hercules, California, USA) and transferred in PVDF membranes. Membranes were probed for FKBP52 (1:1000; R&D MAB4095), GFP (1:1000; Invitrogen G10362), Caspase 3 1:1000; Cell Signaling 9662), Caspase 12 (1:1000; Abcam 62484), GAPDH (1:1000; Proteintech 10494-1), and B-actin (1:1000; Proteintech 66009-1). T22 antibody (a kind gift from Dr. Rakez Kayed (Department of Neurobiology, University of Texas Medical Branch). Blots were incubated in secondary antibodies (1:1,000 dilutions, Southern Biotech) for 1 h and then developed as described above. Molecular weights were determined using the All Blue Prestained Protein Standard (Bio-Rad 1610373). All blots derived from the same experiment and were processed in parallel (Supplementary Figs. 3, 4). Protein analysis was performed using the Image Lab (Bio-Rad) program. In the blots probed for caspase 3, samples affected by bubbles were excluded from the analysis. Cleaved caspase 3 was not detected in these blots.

### Animals

Mice were obtained from our colony at the University of South Florida vivarium, where they are maintained under standard conditions with a 12 h light/dark cycle with free access to food and water and housed up to 5 per cage. All animal experiments were conducted accordingly with the NIH Guide for the Care and Use of Laboratory Animals and the approval from the Institutional Animal Care and Use Committee (IACUC) at the University of South Florida.

rTg4510 mice were bred and used as previously described^46^. For genotyping, 2 mm diameter section of auricular (ear) flap tissue was collected for identification purposes from weanlings. All mice were subsequently genotyped by polymerase chain reaction (PCR) with the use of the following primers: 1) Human Tau cd1F gene F [5′TGA ACC AGG ATG GCT GAG C 3′ and Tau cd5R, 5′TTG TCA TCG CTT CCA GTC C 3′], 2) TET gene F [5′CGC TGT GGC ATT TTAC TTT AG 3′ and TET gene R 5′CAT GTC CAG ATC GAA ATC GTC3′] and housekeeping gene T-cell receptor delta chain (Tcrd) [F, 5′-CAAATGTTGCTTGTCTGGTG-3′, and R, 5′-GTCAGTCGAGTGCACAGTTT-3′] and analyzed using a QIAxcel Advanced system (Qiagen, Valencia, CA, USA).

### Viral injections and tissue processing

At 2.5-month-old, the rTg4510 mice and wild-type mice received bilateral injections into the hippocampus (coordinates = ±3.6, *Y* = − 3.5, and *Z* = + 2.68) with the adeno-associated virus serotype 9 (AAV9)-FKBP52 and AAV9-GFP virus. A stereotaxic equipment (Neurostar GmbH, Tubingen, Germany) was used to deliver 2 μL of 1 × 1012 particles/mL of the corresponding virus. A total of 16 rTg4510 mice [8 for AAV9-GFP; 8 for AAV9-FKBP52] and 19 wild-type mice [10 for AAV9-GFP; 9 for AAV9-FKBP52] were used for behavioral studies performed at 5.5 months of age (allowing 3 months of viral expression). After behavior, half of the animals were used for electrophysiological recordings and the other half for biochemical and immunohistochemical analyses.

Two weeks after completion of behavioral studies, mice were sacrificed, and brains were collected after a cardiac perfusion using 0.9% saline. The right hemisphere of the hippocampi was dissected and snap frozen for biochemical analysis and stored at −80 °C until processed. The left side was fixed overnight in 4% paraformaldehyde for immunohistochemical and stereological analyses. Next day, brains were transferred to 10 % sucrose followed by a change of 20% and 30% sucrose gradients every 24 h. Tissue was then sectioned at 25 μm (immunostaining) and 50 μm (stereology) thickness using a sliding microtome.

### AAV production and purification

The AAV9-GFP and AAV9-FKBP52 plasmids were constructed in our laboratory by subcloning the respective cDNA into a pTR12.1-MCS vector. AAV was produced and purified as previously described^57^.

### Electrophysiology recordings

Long-term potentiation (LTP) recordings were performed as previously described in^58^. Briefly, ex vivo hippocampal slices were obtained from 6-month-old mice, 2 weeks after ending behavioral testing. Mice were rapidly decapitated, and brains were quickly removed, oxygenated, and sectioned at 400 µm thickness using a vibratome. Hippocampi were removed, transferred to an artificial cerebrospinal fluid, ACSF, and allowed to recover before placing electrodes. A stimulating electrode was placed in the CA3 (Schaffer collateral pathway) where recordings were obtained from the CA1 pyramidal neurons. Slices were stimulated at a constant voltage over a period of 5–10 min to evoke field EPSPs. After determining the threshold voltage for the field excitatory postsynaptic potentials (fEPSPs), the input–output (I-O) curve of evoked potentials, fEPSPs, was analyzed. LTP was induced by a 200 Hz theta burst stimulation or one train of tetanus stimuli (high-frequency stimulation; 100 Hz, 1 s, repeated after a 1 min interval). fEPSPs, calculated as the initial slope normalized to the averaged baseline slope of individual slices, were recorded for 1 h after high-frequency stimulation. I–O relations for field potentials was measured by applying increasing voltage intensities (0.5 mV) until reaching maximum fEPSP amplitude. Data presented were obtained from tau transgenic mice AAV9-control, *N* = 23 slices and AAV9-FKBP52, *N* = 12 slices.

### Immunohistochemistry and tissue staining

Tissue was stained free floating as previously described^22,25^ using the following antibodies: AT8 (S202/T205; 1:5000, Thermo Fisher Scientific ENMN1020B), tau (1:100000, Aligent A002401-2), AT180 (pT231; 1:300, AnaSpec 55313-025) and pT181 (1:2000, AnaSpec 54960) with corresponding secondary antibodies (Southern Biotech, 1:3000). For stereology, tissue sections were stained using NeuN-Biotin (1:3000, EMD Millipore MAB377B) and counterstained with 0.05% cresyl violet (Nissl)^22^. Gallyas silver staining was performed as previously described^56^.

### Tissue imaging and quantification

Stained tissue was imaged using a digital slide scanning microscope, Axio Scan.Z1 (ZEISS Microscopy, Munich, Germany). Regions of interest and densitometric analysis were performed using Nearcyte analysis software (Nearcyte.org), as we have done previously^56,59^. The results from this analysis were analyzed using GraphPad Prism.

### Open field

Individual mice were place in the center of a box and allowed to explore for 10 min. Time spent in the center and corners as well as the total distance traveled were monitored and analyzed using the Any-maze video tracking software (www.anymaze.com).

### Fear conditioning and extinction

Each mouse was placed inside a closed chamber and habituated with a white noise (70 decibels) for 1 min before tone presentation. On day 1, for associative fear learning or conditioning, mice were exposed to one-30 s tone only followed by five-foot shocks (0.5 s at 0.5 mA) paired with a 30-s tone. On day 2, extinction phase, animals were placed in the same chamber and exposed eight-30 s tone without a shock. Freezing (lack of movement) during tone presentation was scored by a blind observer to the experiment.

### Radial-arm water maze (RAWM)

The RAWM was used to test for spatial learning and memory deficits in the rTg4510 mice using as described in^60^. The RAWM contained six identical metal arms inside a circular black pool filled with water with an escape platform at the end of one arm. This arm (goal) was maintained in the same location during the trial for each given mouse. On day 1, each mouse was allowed 60 s to locate the platform. Each mouse was tested for a total of 12 trials rotating visible and a hidden platform. On day 2, mice were tested for 12 trials but now leaving the platform hidden during the entire test. Next day, the platform was moved to another location (new goal arm) where mice received 12 additional trials. Entry to an incorrect arm or not entering to any arm within 15 s was counted as an error. The experimenter was blinded to the study and manually scored the number of errors.

### Statistical analysis

Data distributions were tested using Shapiro–Wilks’s test. Group differences were analyzed by one-way ANOVA [dot blot], two-way ANOVA [Open field, tissue stereology], repeated measures two-way ANOVA [ThT Fluorescence, LTP, RAWM], and Student’s *t* test [tissue staining] utilizing GraphPad PRISM version 8.0 (GraphPad Software, San Diego, CA, USA). Significant group difference determined by ANOVA was followed by Bonferroni correction post hoc analysis comparing individual means of experimental groups or genotypes. A difference was considered significant if *p < 0.05, **p < 0.01, and ***p < 0.001 as described in figure legends. Data presented in the figures represent the standard error of the mean (±SEM).

### Reporting summary

Further information on experimental design is available in the Nature Research Reporting Summary linked to this paper.

## Supplementary information

Reporting summary checklist

Supplementary Information

## Data Availability

The datasets used and/or analyzed during the current study are available from the corresponding author on reasonable request.
