# Supplementary material for: FKBP52 overexpression accelerates hippocampal-dependent memory impairments in a tau transgenic mouse model
Source: NPJ Aging Mech Dis. 2021 May 3;7:9. doi: 10.1038/s41514-021-00062-x (PMC8093247; doi:10.1038/s41514-021-00062-x)
Supplement: Supplementary file 1 — Reporting summary checklist [file 41514_2021_62_MOESM1_ESM.pdf]

nature research

Corresponding author(s): Laura J. Blair

Last updated by author(s): January/27/2021

Reporting Summary

Nature Research wishes to improve the reproducibility of the work that we publish. This form provides structure for consistency and transparency in reporting. For further information on Nature Research policies, see our Editorial Policies and the Editorial Policy Checklist.

Statistics

For all statistical analyses, confirm that the following items are present in the figure legend, table legend, main text, or Methods section.

n/a	Confirmed	
☐	☒	The exact sample size (*n*) for each experimental group/condition, given as a discrete number and unit of measurement
☐	☒	A statement on whether measurements were taken from distinct samples or whether the same sample was measured repeatedly
☐	☒	The statistical test(s) used AND whether they are one- or two-sided<br>*Only common tests should be described solely by name; describe more complex techniques in the Methods section.*
☒	☐	A description of all covariates tested
☐	☒	A description of any assumptions or corrections, such as tests of normality and adjustment for multiple comparisons
☒	☐	A full description of the statistical parameters including central tendency (e.g. means) or other basic estimates (e.g. regression coefficient) AND variation (e.g. standard deviation) or associated estimates of uncertainty (e.g. confidence intervals)
☒	☐	For null hypothesis testing, the test statistic (e.g. *F*, *t*, *r*) with confidence intervals, effect sizes, degrees of freedom and *P* value noted<br>*Give P values as exact values whenever suitable.*
☒	☐	For Bayesian analysis, information on the choice of priors and Markov chain Monte Carlo settings
☒	☐	For hierarchical and complex designs, identification of the appropriate level for tests and full reporting of outcomes
☒	☐	Estimates of effect sizes (e.g. Cohen's *d*, Pearson's *r*), indicating how they were calculated

Our web collection on statistics for biologists contains articles on many of the points above.

Software and code

Policy information about availability of computer code

Data collection	*Provide a description of all commercial, open source and custom code used to collect the data in this study, specifying the version used OR state that no software was used.*
Data analysis	ANY-maze, NearCYTE, pCLAMP, SPSS, Graphpad Prism 8, Microsoft Excel, Adobe illustrator CS5 was used for producing final high quality images.

For manuscripts utilizing custom algorithms or software that are central to the research but not yet described in published literature, software must be made available to editors and reviewers. We strongly encourage code deposition in a community repository (e.g. GitHub). See the Nature Research guidelines for submitting code & software for further information.

Data

Policy information about availability of data

All manuscripts must include a data availability statement. This statement should provide the following information, where applicable:

- Accession codes, unique identifiers, or web links for publicly available datasets
- A list of figures that have associated raw data
- A description of any restrictions on data availability

The datasets used and/or analyzed during the current study are available from the corresponding author on reasonable request.

Field-specific reporting

Please select the one below that is the best fit for your research. If you are not sure, read the appropriate sections before making your selection.

- [x] Life sciences
- [ ] Behavioural & social sciences
- [ ] Ecological, evolutionary & environmental sciences

For a reference copy of the document with all sections, see nature.com/documents/nr-reporting-summary-flat.pdf

Life sciences study design

All studies must disclose on these points even when the disclosure is negative.

Sample size	For the animal studies, the sample size was based on prior studies with this strain of mice using intracerebral injections of AAV for similar types of analysis.
Data exclusions	Outliers were identified using the Robust regression and Outlier removal (ROUT) method with PRISM software. Only the outliers due to experimental mistakes were excluded in the analysis.
Replication	For recombinant studies, experiments were repeated at least three times.
Randomization	Animals were randomly allocated to experimental groups
Blinding	Investigators were blinded during group allocation and data collection

Reporting for specific materials, systems and methods

We require information from authors about some types of materials, experimental systems and methods used in many studies. Here, indicate whether each material, system or method listed is relevant to your study. If you are not sure if a list item applies to your research, read the appropriate section before selecting a response.

Materials & experimental systems

n/a	Involved in the study	
☐	☒	Antibodies
☒	☐	Eukaryotic cell lines
☒	☐	Palaeontology and archaeology
☐	☒	Animals and other organisms
☒	☐	Human research participants
☒	☐	Clinical data
☒	☐	Dual use research of concern

Methods

n/a	Involved in the study	
☒	☐	ChIP-seq
☒	☐	Flow cytometry
☒	☐	MRI-based neuroimaging

Antibodies

Antibodies used	Antibodies for WB: FKBP52 (1:1000; R&D MAB4095), GFP (1:1000; Invitrogen G10362), and B-actin (1:1000; Proteintech 66009-1). T22 antibody was a gift from Dr. Rakez Kayed (University of Texas Medical Branch). All secondary antibodies were used at 1:1,000 dilutions (Southern Biotech).<br>Antibodies used in tissue sections: AT8 (S202/T205; 1:5000, Thermo Fisher ENMN1020B), tau (1:100000, Aligent A002401-2), AT180 (pT231; 1:300, AnaSpec 55313-025) and pT181 (1:2000, AnaSpec 54960) with corresponding secondary antibodies (Southern Biotech, 1:3000).
Validation	Commercially available antibodies used as recommended by the manufacturer, except for T22, which was received from Dr. Rakez Kayed and validated in DOI: 10.1096/fj.11-199851.

Animals and other organisms

Policy information about studies involving animals; ARRIVE guidelines recommended for reporting animal research

Laboratory animals	Six month-old males and females were used in this study. Bi-transgenic rTg4510 (129S6/FVB/N) mice were generated by crossing the 4510-responder line (carries a human MAPT P301L cDNA downstream of a tetracycline operon–responsive element) to an activator line (expresses a tetracycline-controlled transactivator, tTA, under the control of a CaMKIIα promoter).
Wild animals	*Provide details on animals observed in or captured in the field; report species, sex and age where possible. Describe how animals were caught and transported and what happened to captive animals after the study (if killed, explain why and describe method; if released, say where and when) OR state that the study did not involve wild animals.*

Field-collected samples	*For laboratory work with field-collected samples, describe all relevant parameters such as housing, maintenance, temperature, photoperiod and end-of-experiment protocol OR state that the study did not involve samples collected from the field.*
Ethics oversight	All animal experiments were carried out accordingly with the NIH Guide for the Care and Use of Laboratory Animals and approved by the USF Institutional Animal Care and Use Committee (IACUC).

Note that full information on the approval of the study protocol must also be provided in the manuscript.
