# Supplementary material for: FKBP52 overexpression accelerates hippocampal-dependent memory impairments in a tau transgenic mouse model
Source: NPJ Aging Mech Dis. 2021 May 3;7:9. doi: 10.1038/s41514-021-00062-x (PMC8093247; doi:10.1038/s41514-021-00062-x)
Supplement: Supplementary file 2 — Supplementary Information [file 41514_2021_62_MOESM2_ESM.pdf]

Supplementary Information for

FKBP52 overexpression accelerates hippocampal-dependent memory impairments in a tau transgenic mouse model

Marangelie Criado-Marrero[1], Niat T. Gebru[1], Lauren A. Gould[1], Danielle M. Blazier[1], Yamile Vidal Aguiar[1], Taylor M. Smith[1], Salma S. Abdelmaboud[1], Lindsey B. Shelton[1], Xinming Wang[2], Jan Dahrendorff[1], David Beaulieu-Abdelahad[1], Chad A. Dickey[1,$], Laura J. Blair[1,3,*]

[1]Department of Molecular Medicine, Morsani College of Medicine, USF Health Byrd Alzheimer's Institute, University of South Florida, Tampa, FL 33613, USA

[2]Department of Molecular Pharmacology and Physiology, College of Medicine, USF Health Byrd Alzheimer's Institute, University of South Florida, Tampa, FL 33613, USA.

[3]Research Service, James A Haley Veterans Hospital, 13000 Bruce B Downs Blvd, Tampa, FL 33612, USA

[$]Deceased

*Corresponding author

Email: laurablair@usf.edu

This PDF file includes:

Supplementary material: Figures S1 to S4

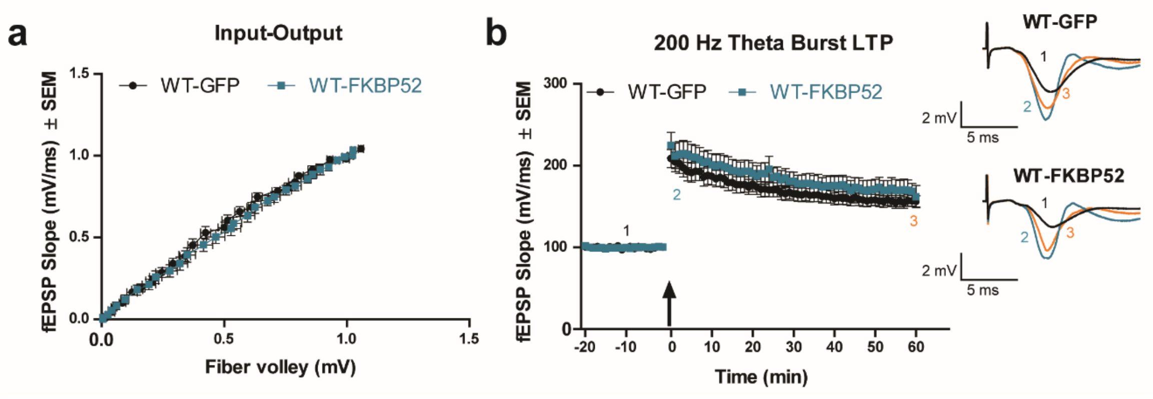

Supplementary Figure 1. **FKBP52 overexpression did not affect hippocampal LTP in WT mice. a** The Input-Output (I-O) curve was calculated to evaluate pre- and post-synaptic excitatory function. The I-O curve compares the fEPSP slope (mV/ms) versus the fiber volley amplitude (mV). **b** LTP was measured in ex vivo hippocampal slices from WT mice infused with AAV9-GFP or AAV9-FKBP52. Stimulating electrode was positioned in the Schaffer collaterals (between CA3 and CA1) and recordings were obtained in the CA1 pyramidal neurons. A 200Hz Theta Burst stimulation was given to induce LTP (indicated by the arrow). Following this, field EPSPs were measured for 60-minutes. Representative traces are shown as: 1 (black) indicates baseline, 2 (teal) indicates initial early LTP in the first minute following HFS, and 3 (orange) indicates late LTP in the last 60 minutes of recording. Data analyzed by repeated measures two-way ANOVA. Data is shown in standard error of the mean (± SEM). n = 27 for WT-GFP and n = 23 WT-FKBP52. AAV9, adeno-associated virus serotype 9; fEPSP = field excitatory postsynaptic potential; LTP, long-term potentiation; min = minutes; mV, millivolts; ms, milliseconds; Hz, hertz.

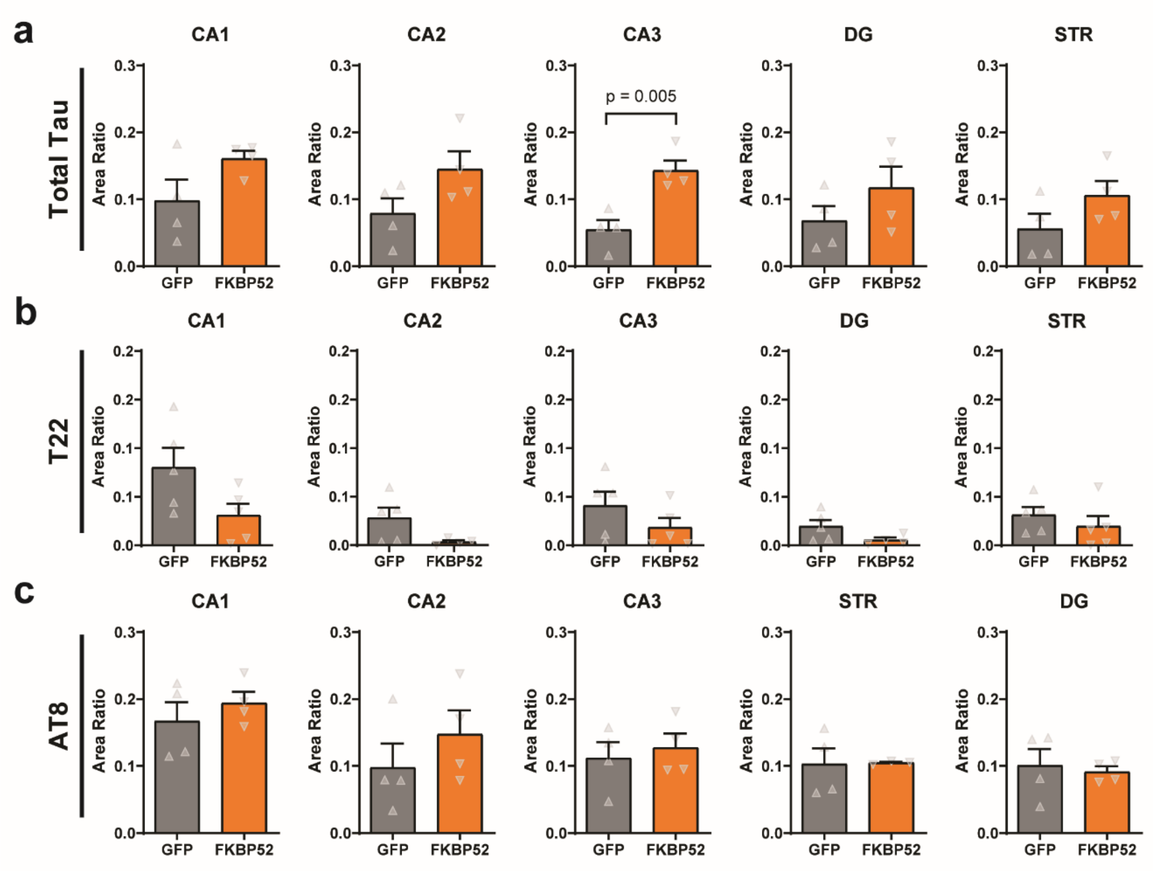

Supplementary Figure 2. Total tau levels were increased in CA3 of rTg4510-FKBP52 mice. Hippocampal slices were obtained from rTg4510 mice injected with AAV9-GFP and AAV9-FKBP52. **a** Quantification of tau levels using tau (DAKO) antibody. **b** Quantification of oligomeric tau levels using T22 antibody. **c** Quantification of pS202/T205 levels (AT8 antibody) in the hippocampi. Protein levels were analyzed by unpaired t-test, where statistical significance is considered by *$p < 0.05$ Results represented as standard error of the mean (± SEM); GFP, n = 4; FKBP52, n = 4).

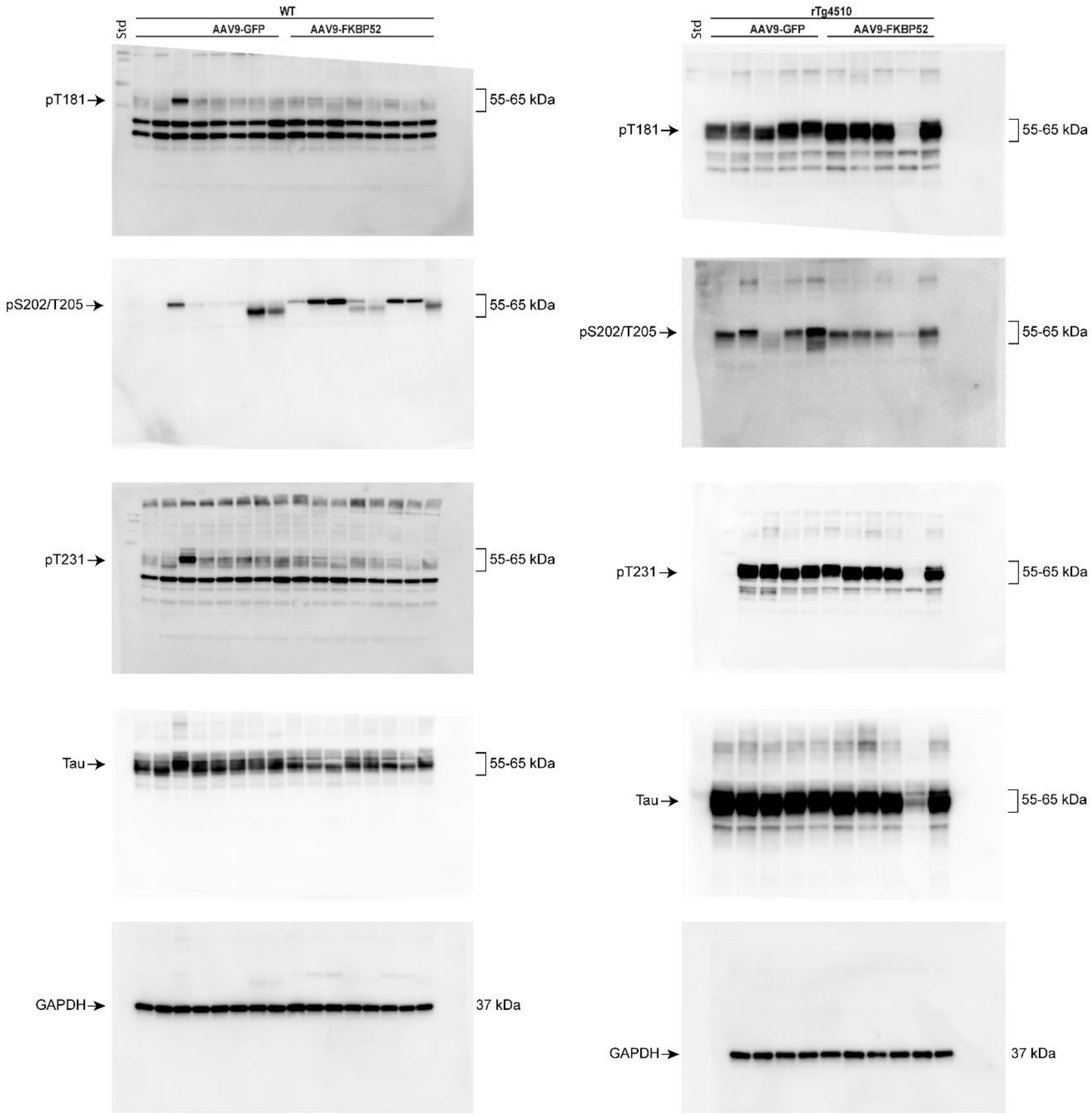

+

Supplementary Figure 3. Western blot membranes for total tau and phospho-tau species. Western blot membranes of soluble fractions for total tau and tau phosphorylated species including pT181, pS202/pT205 (AT8), and pT231 (AT180). Hippocampal tissue from WT and rTg4510 mice expressing either AAV9-GFP or AAV9-FKBP52 was used in these blots. Membranes were run in parallel and derived from the same experiments. Quantification of protein was performed using Image Lab (Bio-Rad) software. Total protein was normalized to GAPDH. WT-GFP (n = 8), WT-FKBP52 (n = 8), rTg4510-GFP (n = 5), rTg4510-FKBP52 (n = 5). AAV9, adeno-associated virus

serotype 9; GFP, green fluorescent protein; GAPDH, Glyceraldehyde 3-phosphate dehydrogenase; WT, wild-type; Std, Protein Standards.

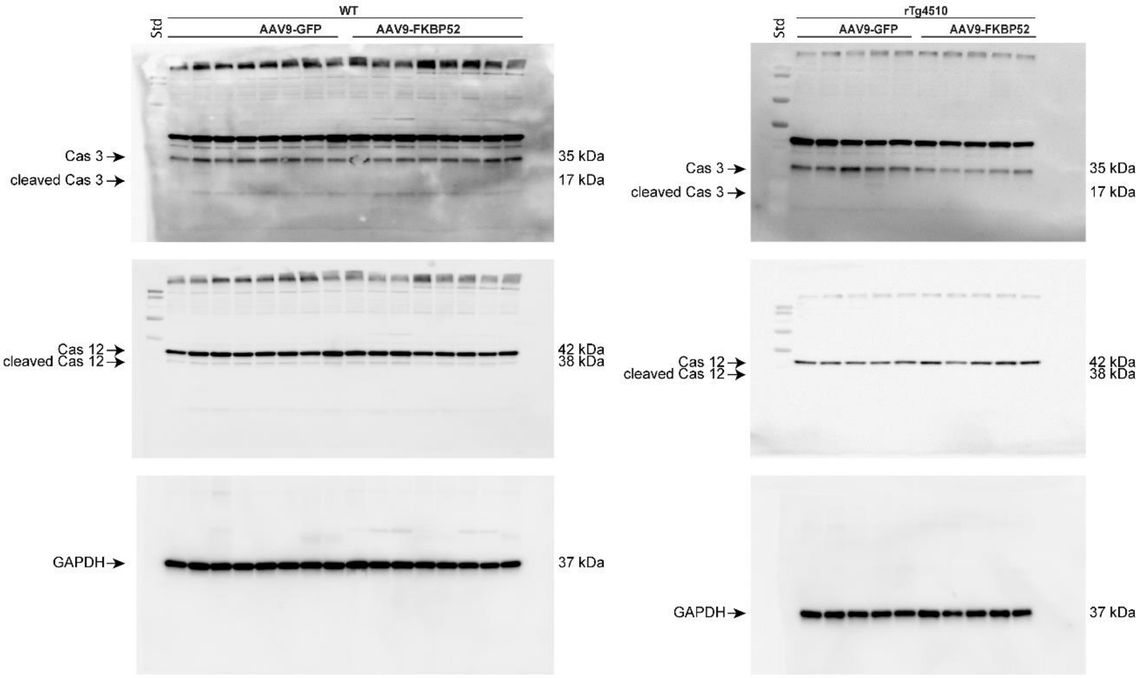

Supplementary Figure 4. Western blot membranes for caspase 3 and caspase 12. Membranes were run in parallel and derived from the same experiments. Quantification of protein was performed using Image Lab (Bio-Rad) software. Total protein was normalized to GAPDH. WT-GFP (n = 8), WT-FKBP52 (n = 8), rTg4510-GFP (n = 5), rTg4510-FKBP52 (n = 5). AAV9, adeno-associated virus serotype 9; GFP, green fluorescent protein; GAPDH, Glyceraldehyde 3-phosphate dehydrogenase; WT, wild-type; Cas, caspase; kDa, kilodalton; Std, Protein Standards.
